# Transcriptomic Analysis in Marine Medaka Gill Reveals That the Hypo-Osmotic Stress Could Alter the Immune Response via the IL17 Signaling Pathway

**DOI:** 10.3390/ijms232012417

**Published:** 2022-10-17

**Authors:** Rong Li, Jiaqi Liu, Chi Tim Leung, Xiao Lin, Ting Fung Chan, William Ka Fai Tse, Keng Po Lai

**Affiliations:** 1 Key Laboratory of Environmental Pollution and Integrative Omics, Education Department of Guangxi Zhuang Autonomous Region, Guilin Medical University, Huan Cheng North 2nd Road 109, Guilin 541004, China; 2 Department of Chemistry, City University of Hong Kong, Hong Kong SAR, China; 3 Department of Psychiatry, Icahn School of Medicine at Mount Sinai, New York, NY 10029, USA; 4 State Key Laboratory of Agrobiotechnology, School of Life Sciences, The Chinese University of Hong Kong, Hong Kong SAR, China; 5 Laboratory of Developmental Disorders and Toxicology, Center for Promotion of International Education and Research, Faculty of Agriculture, Kyushu University, Fukuoka 819-0395, Japan; 6 State Key Laboratory of Marine Pollution, City University of Hong Kong, Hong Kong SAR, China

**Keywords:** cell cycle, p53 pathway, FoxO signaling, immune system, gill mucosal immunity

## Abstract

Fish gills are the major osmoregulatory tissue that contact the external water environment and have developed an effective osmoregulatory mechanism to maintain cellular function. Marine medaka (*Oryzias melastigma*) has the ability to live in both seawater and fresh water environments. The present study performed a seawater (SW) to 50% seawater (SFW) transfer, and the gill samples were used for comparative transcriptomic analysis to study the alteration of hypo-osmotic stress on immune responsive genes in this model organism. The result identified 518 differentiated expressed genes (DEGs) after the SW to SFW transfer. Various pathways such as p53 signaling, forkhead box O signaling, and the cell cycle were enriched. Moreover, the immune system was highlighted as one of the top altered biological processes in the enrichment analysis. Various cytokines, chemokines, and inflammatory genes that participate in the IL-17 signaling pathway were suppressed after the SW to SFW transfer. On the other hand, some immunoglobulin-related genes were up-regulated. The results were further validated by real-time qPCR. Taken together, our study provides additional gill transcriptome information in marine medaka; it also supports the notion that osmotic stress could influence the immune responses in fish gills.

## 1. Introduction

Osmoregulation is a critical biological mechanism for various cellular functions such as defining the cell shape and maintaining the intracellular osmolality [1]. Euryhaline fish can reside in both fresh water and seawater. The fish encounter totally opposite osmotic challenges in the two media. In fresh water, the fish deal with osmotic water uptake and loss of salts and vice versa in seawater. Fishes develop an osmoregulatory mechanism to regulate fluid and ion homeostasis to maintain a constant body osmolality in their lives. Fish gills are one of the primary ion-osmoregulatory tissues that constitute about 50% of the surface area of the fish interface with the surrounding medium, and thus develop specific osmoregulatory mechanisms [2]. The remodeling of gill tissues upon salinity changes has been reported [3,4].

Marine medaka (*Oryzias melastigma*) has the ability to live in both seawater and fresh water, which indicates that its gill could modify the ion and water homeostasis for the ion loss and water gain during hypo-osmotic stress. Due to its ability to live in seawater and well-known genome, it became a common fish model to study the marine pollution in the past few years [5,6,7]. Recently, we noticed that an omics paper on this ecotoxicity model was published. The study reported the gill and liver transcriptomics data of the four-month-acclimated fish in different salinities since the embryonic stage [8]. The authors aimed to understand the responses of *O. melastigma* under chronic salinity stress by various developmental and histochemical parameters and RNA-Seq. However, the result did not address the early hypo-osmotic responses in an adult fish. It is known that the osmoregulatory mechanism can be divided into three phases; namely, the osmosensors, signal transducers, and effectors [9]. Taking the gill as an example, the sensors in the gill cells detect the change of external osmolality and thus lead to the stimulation of various signaling molecules, and finally induce effectors to compensate for the osmotic challenge. The whole process can be completed in seven to fourteen days; and the sensing and transduction can be achieved from a few minutes to six hours. Signaling events such as the phosphorations of MAPK, JNK, and ERK and the upregulation of the osmotic stress transcription factor (Ostf1) were identified [10,11,12,13]. Lastly, the effectors, such as ion transporters, could modulate their mRNA and protein expressions afterwards, which could be achieved in a week [14]. The prolonged osmotic stress in the published study [15] identified the differentially expressed genes (DEGs) in the fish gill and liver raised from different osmotic salinities, and thus, did not cover the early phases of the osmotic regulation that this study is reporting.

Various environmental factors, such as temperature, salinity change, and oxygen levels, are found to affect the immune system in fishes [15]. Gills contain the large mucosal surfaces that protect the fish from pathogen entry [16]; it forms the gill-associated lymphoid tissue that is composed of various immune cells such as lymphocytes, macrophages, and antibody-secreting cells [17,18,19]. Mucus contains microbiota that could help in osmoregulation and protect the host from infection [20,21]. More importantly, the amount of the mucus production can be affected by water hardness and salinities [22], and the mucus production is higher in the gill surrounding area than the skin sites [23]. These findings further suggest the relationship between salinity and gill mucosa barrier in fishes.

Studies have demonstrated that the osmotic stress could influence the immune system in fishes; for example, an increase of plasma immunoglobulin (Ig) M level in seabream, and white blood cell count in rainbow trout were found after hyper-osmotic stress [24,25]. Numerous studies have been done in immune organs such as the kidney and spleen in fishes. However, how the salinity affects such system in gills is not well-known. Different immune proteins such as toll-like receptor 2 (TLR2) and interleukin-1 receptor type 2 (IL-1R2) are highly expressed in gill cells [26], and a report of eel gill has demonstrated that the immune system could be affected by osmotic stress [27]. In this study, we conducted gill transcriptomic analysis from seawater-acclimated marine medaka (control group; SW), and a group that was transferred from seawater to 50% seawater for seven days (hypo-osmotic stress group; SFW) to gain insight into the immune responses in gill of marine medaka upon hypo-osmotic stress.

## 2. Results

**Figure 1:**
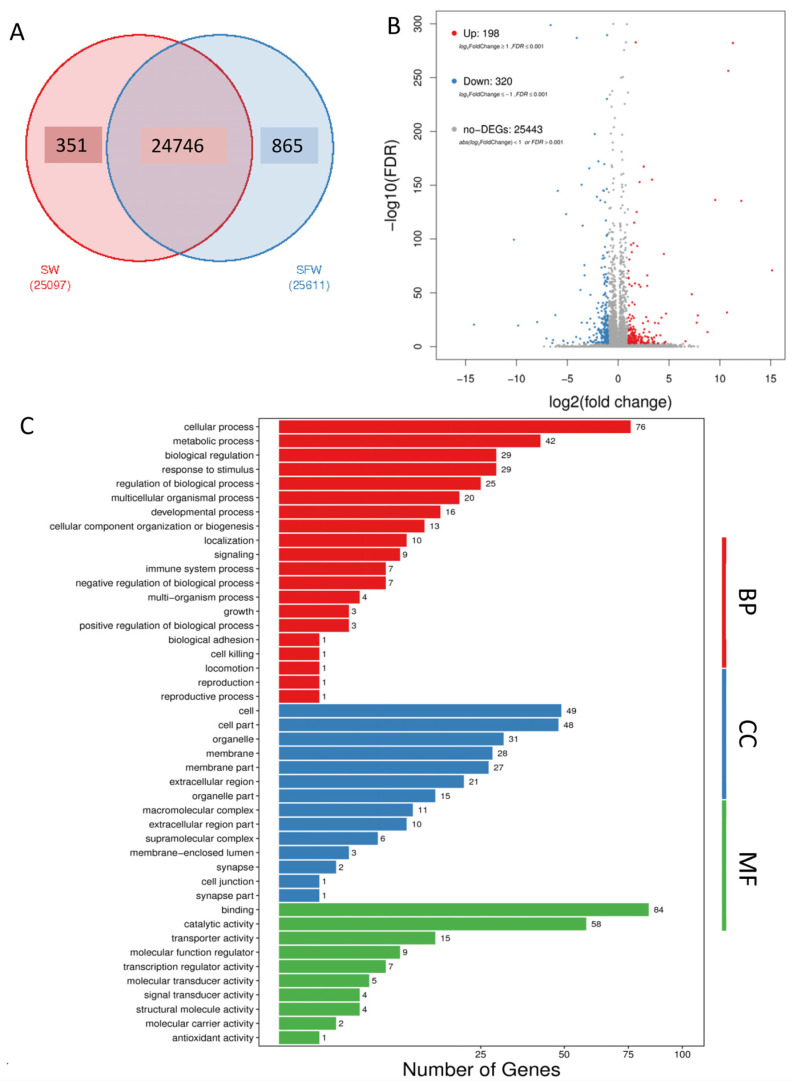
Identification of differentiated expressed genes (DEGs) in medaka gill after seawater (SW) to 50% seawater transfer (SFW). (**A**) Venn diagram showing the comparison between SW and SFW. There were 24,746 transcripts identified as common counts. (**B**) Volcano plot showing 198 up-regulated DEGs and 320 down-regulated DEGs in the SW/SFW group. Red dots indicate the up-regulated genes, while blue represent the down-regulated. Non-DEGs were marked as grey dots. (**C**) Gene Ontology (GO) of DEGs after the transfer. The enriched GO terms were classified into biological process (BP, red), cellular component (CC, blue), and molecular function (MF, green). The top enriched terms were cellular process in BP; cell in CC; and binding in MF.

A total of 25,962 genes were identified, in which 24,746 genes were commonly found in both SW and SFW groups (Figure 1A). The sequencing information such as the mapping ratio, and clean read data are summarized in Supplementary File S1. Five hundred and eighteen DEGs were identified after the transfer, including 198 genes which increased their expression, and 320 genes decreased in the gill of the SFW group (Figure 1B, Supplementary File S2). The DEGs were then subjected to GO and KEGG enrichment analysis to understand the biological roles and functions of the DEGs. Enriched GO terms were grouped into the biological process (BP), cellular component (CC), and molecular functions (MF). The top enriched terms in BP (red color) were the cellular process, followed by the metabolic process. In addition, biological adhesion and immune system process were also identified. Furthermore, cell and binding were the top enriched terms in CC (blue) and MF (green), respectively (Figure 1C, Supplementary File S3). The GO relationship network among the enriched terms was further generated and shown in Supplementary File S4.

**Figure 2:**
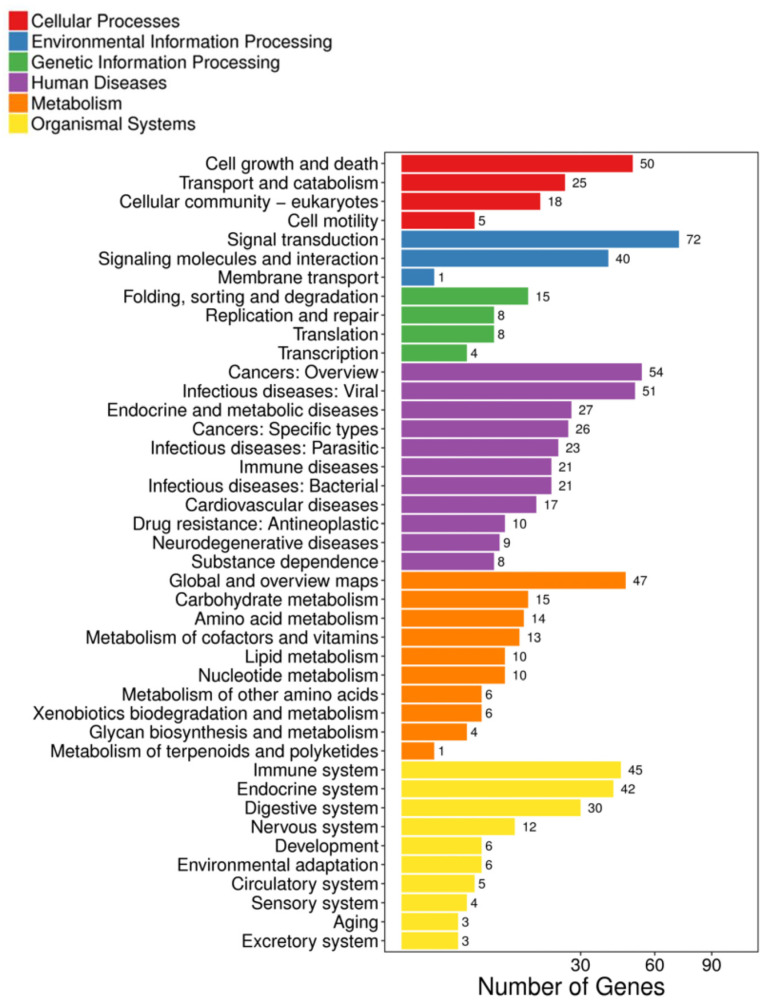
Pathway classification of DEGs in SW/SFW group. *X*-axis represents the number of DEGs, and the *Y*-axis indicates the functional classification of KEGG that has been further classified into seven categories: cellular processed, environmental information processing, genetic information processing, human disease, metabolism, and organismal systems. The signaling transduction under the environmental information processing was enriched by the highest DEGs (72 genes). Immune system (45 genes) was the top enriched term in organismal systems.

**Figure 3:**
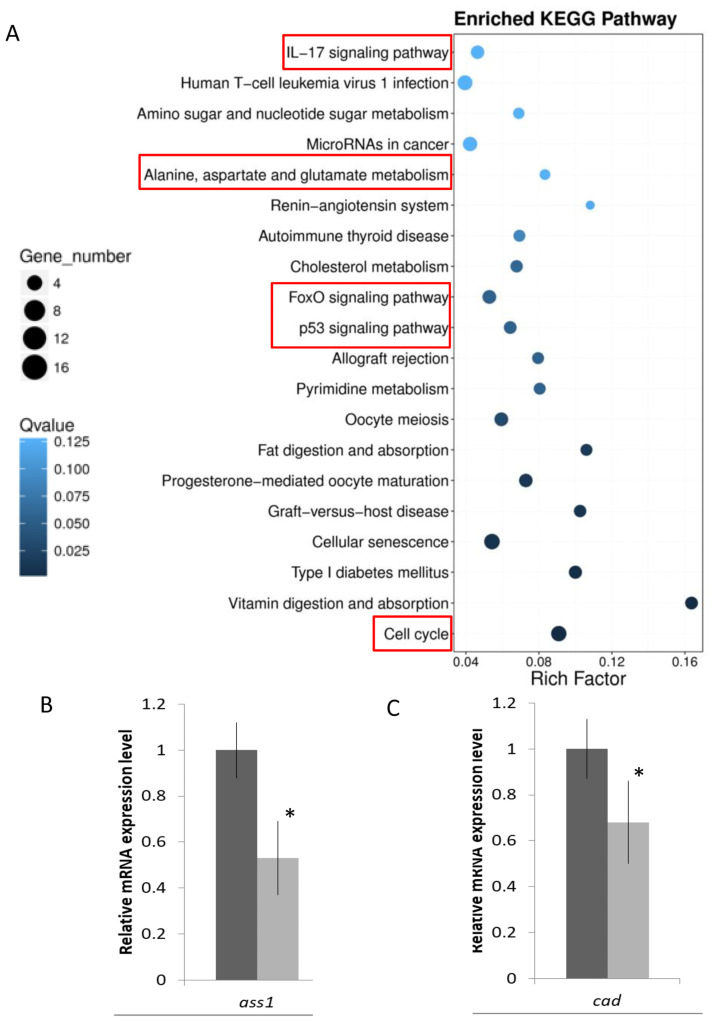
Enriched KEGG pathways in SW/SFW group. (**A**) Pathway functional enrichment of DEGs in SW/SFW group. *X*-axis represents the enrichment factor, while *Y*-axis indicates the enriched pathways. The color indicated the q-value; the blue color represented the lower value, while the white color indicated the higher value. In addition, the point size indicated the number of DEGs; bigger dots represented more genes. The greater the rich factor, the more significant enrichment. IL17 signaling pathway, alanine, aspartate, and glutamate metabolism, FoxO signaling pathway, p53 signaling pathway, and cell cycle (red boxes) were found to be enriched in the SW/SFW group. (**B**,**C**) Validations of the mRNA gene expression levels of two selected alanine and glutamate metabolism related genes (*ass* and *cad*) transcripts by real time qPCR. Results matched with the sequencing data. *n* = 5; mean ± s.e., * indicated *p* < 0.05.

KEGG pathway analysis was further performed to show the functional enrichment from the DEGs. Different enriched terms were spotted: cell growth and death were found in the cellular processes; signal transduction and membrane transport in environmental information processing; folding, sorting, and degradation in genetic information processing; cancers in human diseases; amino acid metabolism and lipid metabolism in metabolism; and immune system and endocrine system in organismal systems (Figure 2). When the data was shown at a higher resolution, more specific pathways such as the IL-17 signaling pathway, alanine, asparate, and glutamate metabolism, FoxO signaling pathway, p53 signaling pathway, and cell cycle were spotted (Figure 3A, Supplementary File S5). Validations of selected genes in the above pathways by real-time qPCR were performed. The relative mRNA expression level of argininosuccinate synthase 1 (*ass1*), and carbamoyl-phosphate synthetase 2, aspartate transcarbamylase, and dihydroorotase (*cad*), which are responsible for amino acid and pyrimidine metabolism, were decreased (Figure 3B,C).

**Figure 4:**
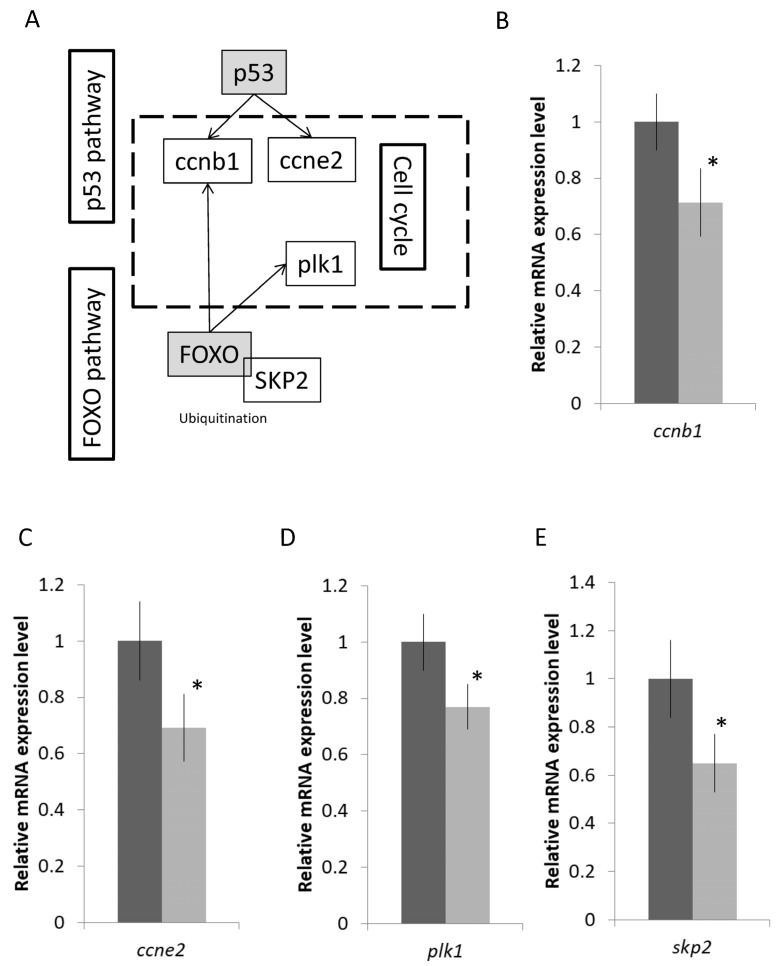
Hypo-osmotic stress influences the FoxO, p53 pathways, and cell cycle in gill cells. (**A**) The relationship among FoxO, p53, and cell cycle. (**B**–**E**) Validations of the mRNA gene expression levels of the selected transcripts by real time qPCR. Down regulation of the selected transcripts (*ccnb1*; *ccne2; plk1*; and *skp2*) in the pathways. Results matched with the sequencing data. *n* = 5; mean ± s.e., * indicated *p* < 0.05.

**Figure 5:**
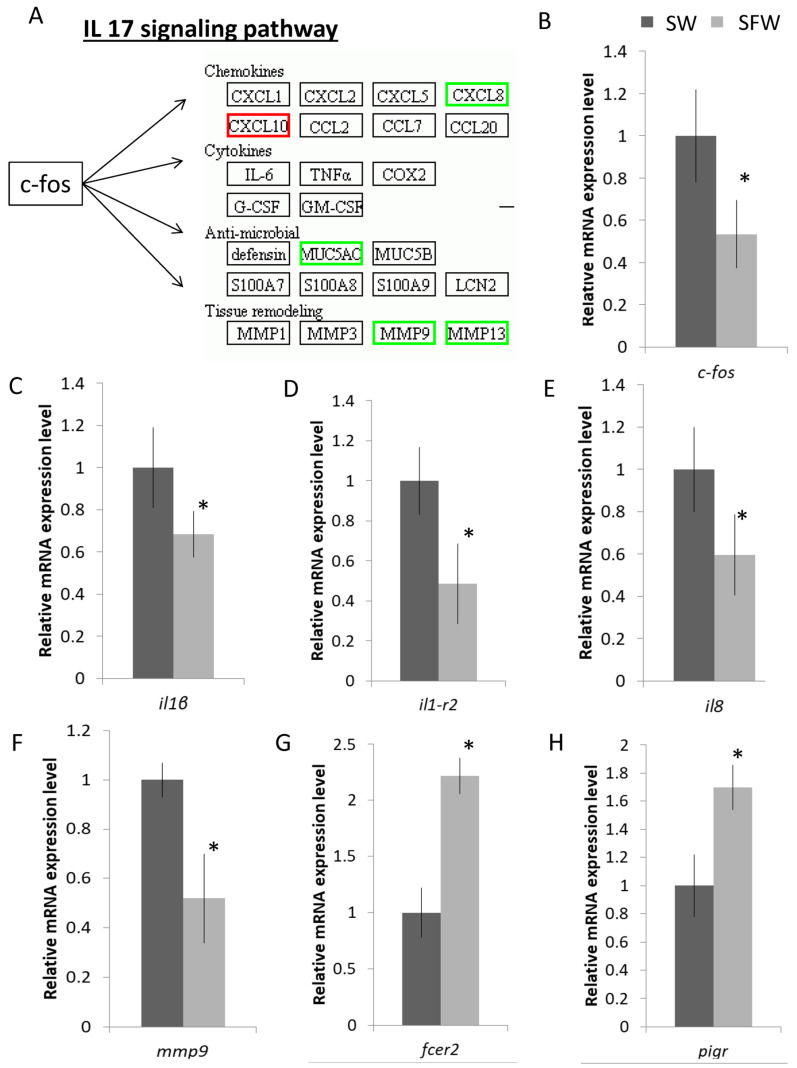
Hypo-osmotic stress influences the immune system in gill cells. (**A**) IL-17 signaling pathway was enriched in the SW/SFW group; green indicated the reduced transcripts, while red indicated the up-regulated transcript. The transcription factor, *c-fos,* could induce various down-stream targets. (**B**–**H**) Validations of the mRNA gene expression levels of the selected transcripts by real time qPCR. Down-regulation of the selected immune-related genes (transcription factor *c-fos*; interleukin-related genes (*il1-r2*; *il1b*; and *il8*); tissue remodelling gene, *mmp9*) and up-regulation of the selected immunoglobulin-related receptors (*fcer2;* and *pigr*) after the transfer. Results matched with the sequencing data. *n* = 5; mean ± s.e., * indicated *p* < 0.05.

Genes that participated in the FoxO and p53 pathways that are related to the cell cycle were further validated as well (Figure 4A). The mRNA expressions of S-phase kinase-associated protein 2 (*skp2*), polo-like kinase 1 (*plk1*), cyclin E2 (*ccne2*), and cyclin B1 (*ccnb1*) were reduced after the transfer (Figure 4B–E). Lastly, we focused on the enriched immune-related IL-17 pathway that includes the core activator protein 1 transcription factor, *c-fos,* and its downstream effectors such as chemokines, cytokines, anti-microbial, and tissue remodelling. Genes labelled in the green boxes were suppressed after the fresh water transfer, while the one in the red box was the induced one (Figure 5A). The IL-17 signaling pathway involves the activator protein 1 (AP-1) transcription factor, *c-fos*, which could activate a large number of downstream effectors such as chemokines, cytokines, and inflammatory genes (Figure 4A). The mRNA expression levels of selected genes such as *c-fos*, *il-1β*, *il1-r2*, *il8*, and *mmp9* were decreased after the SW to SFW transfer (Figure 5B–E). Additionally, numerous immunoglobulin-related transcripts were induced upon hypo-osmotic stress in this study. For example, Fc epsilon receptor II (*fcer2*) and polymeric immunoglobulin receptor (*pigr*) were upregulated (Figure 5G–H). Lastly, IPA analysis was performed to have an overview of the possible bio-functions and related diseases of the DEGs. Significant changes of the immune-related terms were spotted. It was noticed that the IL-1β was commonly found in the pathways that matched with other analysis (Table 1). The full list of the terms can be referred to in Supplementary File S6.

## 3. Discussion

Our findings identified various biological processes upon hypo-tonic stress in marine medaka gill. The enrichment of cell-cycle-related genes have been identified in other fishes under osmotic stress [28,29,30]. A study in fish esophageal cells showed the occurrence of cell proliferation or cell death during the osmotic challenges [31]. In addition, the shift of the seawater or freshwater type of gill mitochondria-rich cells upon osmotic stress were achieved by cellular proliferation and apoptosis that are controlled by the cell cycle regulators [32]. FoxO transcription factors are known as critical regulators of the cellular stress response and act as the redox regulators [33]. The induction of the FoxO signaling pathway during osmotic stress was reported in oyster [34]. Additionally, it has been suggested to play roles in extending the life span in *C. elegans* [35]. FOXO proteins are regulated by the ubiquitination of SKP2 and control the cell-cycle-related genes such as PLK1 and CCNB1 [36,37,38]. Here, various cell-cycle-related genes were down-regulated, which further supported the notion that several cell cycle regulators could play roles in modifying cell proliferation, differentiation, and survival during osmotic stress [39]. Furthermore, the p53 pathway is closely related to cell death and cell cycle regulations. A hyper-tonic stress experiment on climbing perch could induce apoptosis and p53 protein expression in gill [32]. Moreover, all these pathways are found to be closely related to hypoxia-induced cell cycle arrest [40]. Studies in killifish and hagfish have demonstrated the relationship between salinity acclimation ability and the hypoxia condition. Changes of plasma osmolality and ion flux rates in gills were found in fishes under hypoxia [41,42]. Furthermore, hypoxia could induce the immune response in fish gill. A study in large yellow croaker showed changes of mRNA expression levels of various chemokines and their receptors via a transcriptome study after hypoxia stress [43]. All these findings further support the notion of the relationships between salinity stress, immune response, and hypoxia.

Fish gills, besides their osmoregulatory function, play important roles in immune response. Since fishes are continuously exposed to the external medium rich in microbiota, they develop various mucosa-associated lymphoid tissues (MALT) to protect themselves from infection. There are four major types of MALT, including the gill, gut, skin, and nasopharynx [44]. The mucosal immune system is composed of innate and adaptive immune cells and molecules to protect the host against pathogens. Mucosal B and T cells and the presence of specialized mucosal antibodies have been identified in teleost fish [45]. B cells, plasma cells and the Igs form the mucosal barriers [46], in which the B cells contribute to only about 1% of total gill leukocytes in the rainbow trout [47]. On the other hand, T cells contribute to 10–20% of the lymphoid cells in gill-associated lymphoid tissue, [48].

Osmotic stress affects immune responses. Osmoregulatory cytokines, such as interleukins (ILs), were reported to play roles in the osmotic stress signaling network in fishes to regulate epithelial responses to salinity changes [49,50,51]. ILs are the inflammatory cytokines and have been reported to respond to osmotic stress [27]. IL-17 belongs to the pro-inflammatory cytokines that are produced by helper T-cells and could induce varies cytokines and chemokines, such as IL-1β and IL-8, suggesting its roles in immune response. Furthermore, IL-17 signaling is found to play important roles in the host’s metabolic activity and participates directly in the induction of metabolism-related genes [52]. In addition, the IL-17 signaling pathway is involved in tissue repair and tumorigenesis [53]. It is known that when the tissue is damaged, the immune Th17 cells produce IL-17 and protect the mucous membranes and epithelial tissues against infection. Additionally, the Th17 cells expand at the mucosal surface to clear the invading microorganisms [54,55]. Furthermore, Th17-cell-associated cytokines are involved in wound healing by modulating the mucosal surfaces [56]. The IL-17 signaling pathway involves the core transcription factor, c-Fos, that could activate effectors such as chemokines, cytokines, and inflammatory genes [57,58]. Among the cytokines’ families, the IL-1 family plays a critical role in the innate immune response, in which the IL-1 receptor is responsible for the fundamental inflammatory response [59]. IL-1β is the most studied member in the IL-1 family. It is a pro-inflammatory cytokine that is secreted by macrophages and could further modify the nature of immune cells [60]. Although osmotic stress (hyper-osmolality) has been recognized as a pro-inflammatory stress to the corneal epithelium [61], the relationship between the immune response and osmoregulation is still not well-described. Nevertheless, some in vitro mammalian cell line studies have demonstrated that osmotic stress could stimulate the IL signaling pathway. For example, a study using the human limbal epithelial cell lines showed a higher IL-1β and IL-8 concentrations and mRNA expressions in the hyperosmolar culture medium-cultured cells [62]. Furthermore, another hyperosmotic stress study in the human corneal epithelia cells showed the induction of IL-1 [61]. Our reduced mRNA expression levels of *il1β* and *il8* under hypo-osmotic stress matched with those reports. Regarding the studies in fishes, a study in tilapia found that acute hypo-osmotic stress could suppress the immune response as indicated by lymphopenia and neutrophilia [63]. Another hypo-osmotic stress study in seabream demonstrated the functional alternations of inflammatory reactions along the brain-gut axis, with the involvement of IL-1β [64]. Moreover, an in vitro study using the renal masses in spotted scat also identified the changes of mRNA expression levels of various pro-inflammatory cytokines, including the *il-1β* upon osmotic stress [65].

Additionally, chemokines play roles in the immunoregulatory and inflammatory processes [66]. The chemokine IL-8 has been reported as the osmo-responsive gene in human epithelial cells [62]. Its mRNA expression level was reduced in gill upon hypo-osmotic stress (Figure 4B,F). Ig are glycoproteins that have antibody activity for immune response. Receptors interacting with the Ig play critical immune reactions in vertebrates [67]. For example, *fcer2* is classified as the low-affinity receptor for IgE, which plays roles in allergy reaction and modulating the IgE serum levels [68,69]. Although it is known that the IgE is unique in mammals [70], *fcer* is identified in the genome of bony fishes such as zebrafish (ENSDARG00000103276.3) and medaka (ENSORLG00000016096.2). The reason for its presence in fishes and corresponding functions are not well-understood. A study in zebrafish demonstrated a cross-reactivity of antibodies against human FcεRIγ and human IgE on the zebrafish high-affinity IgE-like receptor (*fcer1*) [71]. More importantly, stimulation of FcεRI receptor by IgG and other immune mediators is found in mammals [72,73]. This may suggest that the medaka *fcer* may be responsive to other Ig that requires further investigation. In terms of evolution, *pigr* members represent a major step in Fc receptor evolution. They are not found in cartilagous fish and are suggested to have first appeared within bony fishes [67]. In fish, *pigr* bind with various Ig [74] and is highly expressed in fish gill [75]. Our results marked the increased mRNA expression levels of these two receptors which further supports the notion that osmotic stress could influence the immune responses. All these results support the notion that osmotic stress could influence the immune response.

Lastly, various metabolomics studies in fishes have identified that osmotic stress could alter metabolic pathways. A transcriptomic study identified numerous enriched metabolic pathways in eel gill after fresh water to seawater transfer [29]. A catfish study suggested that hyper-osmotic stress could alter amino acid metabolism [76], while glutamate metabolism was enriched in tonguefish [77]. We demonstrated the reduced mRNA expression of *ass1* and *cad*, which are responsible for amino acid and pyrimidine metabolism after the transfer. Moreover, various studies in aquatic lives have identified the changes of amino acid metabolism and immunity together during osmotic challenges. For example, a study in crab found that hyper-osmotic stress could affect energy metabolism, amino acid metabolism, and immunity [78], while glucose metabolism and the immune system were enriched in prawn upon hypo-tonic stress [79].

This study is limited in that we did not demonstrate the presence of the immune cell (Th cell) in the gills via immunohistochemical CD4 staining [80]. The condition of the fish before and during the transfer experiment showed no infections or other obvious abnormalities. Regarding the presence of the immune cells in medaka gills, studies have identified the immune-related proteins, such as B lymphocyte-induced maturation protein-1 and Hepatitis A virus cellular receptor 2, in gills that are responsible for maturation of lymphoid cells [81] and as a T helper 1-specific cell surface protein [82], which support the presence of immune cells in gills.

To summarize, osmotic stress could not only influence the typical well-known water/ion homeostasis and cell-cycle-related genes, but could also modulate the immune system via IL-17 signaling pathway in medaka gill. The underlying osmoregulatory mechanisms among fish ILs, Ig, and their relative receptors must be further studied to fill the immune-osmotic stress knowledge gap in fishes.

## 4. Materials and Methods

### 4.1. Fish Maintenance and Experimental Setup

Six-month-old medaka (*O. melastigma*) was maintained in artificial seawater (Sea Treasure, Tokyo, Japan) at 26 °C. Fifty fish were kept in a 20 L tank for more than one week prior to the transfer experiment. Twenty-five fish were transferred to 50% seawater for seven days as the SFW transfer group. The remaining twenty-five fish were the control group that had gone through the seawater-to-seawater transfer (SW). The density of the SW was measured and confirmed by the refractometer at 1.02 g/cm^2^. The 50% seawater was prepared by diluting the seawater with aerated fresh water that reached the density at 1.01 g/cm^2^. One-third of the rearing water in tanks was changed every three days to maintain good water quality. The tanks were constantly aerated, and the fresh water was prepared by de-chlorinating tap water for at least a week. Fish were fed with brine shrimp (Brine Shrimp Direct, Ogden, UT, USA) once a day and commercial fish feed twice a day (Marubeni, Tokyo, Japan).

### 4.2. Fish Sampling

Twenty medaka from each group were anesthetized by the MS222 (Sigma-Aldrich, Lanham, MD, USA). A pair of gills was collected, and their total RNA was extracted by the TRIZOL reagent (Life Technologies, Carlsbad, CA, USA) for transcriptome sequencing. Briefly, five gills from five individuals were pooled as one biological sample (4 replicates from each condition) for RNA sequencing. The experiments were performed using protocols approved by Kyushu University, Fukuoka, Japan (A19-165-1).

### 4.3. Library Construction and Illumina RNA-seq

For library construction, RNA concentrations were measured using a Qubit RNA Assay Kit on a Qubit 2.0 Fluorometer (Life Technologies, Carlsbad, CA, USA). We used 300 ng total RNA with an RNA Integrity Number > 8 for the library construction (Agilent Technologies, Santa Clara, CA, USA), followed by the qualification using the Agilent 2100 Bioanalyzer system. cDNA libraries were prepared by the TruSeq Stranded mRNA LT Sample Prep Kit (Illumina, San Diego, CA, USA), and the index codes were ligated afterwards to identify the individual samples. Lastly, mRNA was purified from total RNA using poly-T oligo-attached magnetic beads (Illumina, San Diego, CA, USA) before fragmentation. cDNAs were synthesized by applying random oligonucleotides and SuperScript II with DNA polymerase I and RNase H treatment. Overhangs were then blunted by treatment with exonuclease/polymerase, and the 3′-end adenylation and ligation to Illumina PE adaptor oligonucleotides were then performed. DNA fragments with adaptor molecules on both ends were enriched by the Illumina PCR Primer Cocktail with 15 PCR cycle. Products were then purified by the AMPure XP system and quantified by the Agilent Bioanalyzer 2100 system and were then sequenced by the BGISEQ-500 platform. Sequence reads were filtered by the SOAPunke software (v1.5.2, Beijing Genomics Institute, Shenzhen, China) to remove reads with adaptors, > 0.1% unknown bases (N), and low-quality reads (percentage of base that quality is lesser than 20 is greater than 50% in a read). Sequencing reads were mapped to the reference genome using Bowtie2 (v2.2.5, Johns Hopkins University, Baltimore, MD, USA) [83], and the gene expression level was calculated by the RSME software package [84]. Clustering analysis was done by the Mfuzz software (v2.34.0, University of Algarve, Faro, Portugal) with the parameters of c = 12; m = 1.25 [85]. The SW raw data used in this study was extracted from our previously published data [20], and the SFW-transferred fish in this study were obtained from the same patch of the fish. The DEGs between SW/SFW were detected by the PossionDis with fold change ≥ 2.0 and FDR ≤ 0.001 [86]. Gene Ontology (GO) and Kyoto Encyclopedia of Genes and Genomes (KEGG) analysis were then performed by using phyper, a function of R. The false discovery rate (FDR) smaller than 0.01 was defined as significant enriched. The QIAGEN’s Ingenuity^®^ Pathway Analysis Release (2019) (IPA, QIAGEN Redwood City, CA, USA, www.qiagen.com/ingenuity, accessed on 3 September, 2020), was used to delineate the disease and biological functions of the DEGs after the transfer. The parameter was set as the default as described [87].

### 4.4. Reverse Transcription and Quantitative Real-Time PCR

Total RNA was extracted from another new set of transfer experiments with the same setting as mentioned. Five fish (*n* = 5) were used in each group (SW control, and SFW transferred group) for sampling. Purified gill RNA with a ratio of 1.8–2.0 at A260/A280 was used to synthesize cDNA using SuperScript VILO Master Mix (Invitrogen; Life Technologies) by PTC-200 Peltier Thermal Cycler (MJ Research, St. Bruno, QC, Canada). Real-time qPCR was performed by using the Power SYBR Green PCR Master Mix (Applied Biosystems, Waltham, MA, USA) and QuantStudio 3 Real-Time PCR System (Applied Biosystems). Primer sequences were tabulated in Table 2. The relative expression ratio was calculated according to the method described by Pfaffl [88] and the data were normalized using the expression levels of 18S-ribosomal RNA. The existence of primer–dimers and secondary products was checked using melting curve analysis. All data are represented as mean ± s.e. Statistical significance was tested using Student’s *t*-test or one-way analysis (ANOVA), followed by Tukey’s HSD test. Groups were considered significantly different if *p* < 0.05. Normality test was performed using the Kolmogorov–Smirnov normality test in SPSS software.

## Figures and Tables

**Table 1:** IPA analysis (disease and bio-function) of the DEGs in gill upon hypo-osmotic stress. Selected terms were shown.

Disease or Functions Annotation	Activation Z-Score (SW/SFW)	Number of Genes (Interleukins Related)
Recruitment of phagocytes	−2.107	18 (IL1B)
Recruitment of leukocytes	−1.882	23 (IL1B; IL1R2)
Proliferation of immune cell	−0.606	39 (IL1B)
Cell death of immune cell	1.183	36 (IL1B)
Transmigration of phagocytes	0.834	9 (IL1B)
Systemic autoimmune syndrome	0.640	61 (IL1B; IL1R2)
Activation of neutrophils	0.579	10 (IL1B)

**Table 2:** qPCR primers used in the study.

Gene	Primer—F (5′ --> 3′)	Primer—R (5′ --> 3′)
*ass1*	GCAGAAATTTGGCATTCCGGT	GCCGGGTTTTTGGTCATCAAG
*cad*	ACGGGAACACCCAGAAATCC	CAGAGTAGTCGAACTCGCCC
*ccnb1*	TGACTACGACAACCCCATGC	TGAGGATGGCTCGCATGTTT
*ccne2*	CGCTTACTTGGCTCAGGACT	CAGGCTCCATCTGTGACGAA
*cfos*	GACAGCATCAAGTGCCTCCT	CACGTTTGGAAGAGCAAGCC
*fcer2*	GAGGAAGAGATCCAATACTCCTCTG	ACAGCAGGTGATGAAACCATCT
*il1-r2*	TGGATCCAGAGGTGGGGATT	GAGGAACCAGAGTTGGGTGG
*il1β*	GGGCATCAAGGACACCAAAC	GTGAGGGTGCTGAGGTTTCC
*il8*	ACAATAACGGCCTTCGCGTT	GTTGGAAGTTGTGAGGGTGC
*mmp9*	TTATCCTCCTGGTGAGGGCA	CGCCGAAAACAAAGGGGAAG
*pigr*	TGGTCACTCCACTACCCACA	CGCCAACAAGTGAGTGTGAC
*plk1*	GGCTCGCTACTACATGACCC	GTGGCCAAACCAAAGTCACC
*skp2*	CGGGTACAGAGAGAGCCTCA	GTGATAGCAGCGACTCAGGG
*18S*	CCTGCGGCTTAATTTGACCC	GACAAATCGCTCCACCAACT

## Data Availability

The sequencing data from this study have been submitted to the NCBI BioProject (https://www.ncbi.nlm.nih.gov/bioproject, accessed on 10 August 2022) under the accession number PRJNA588335. Other data supporting the result of this article are included within the article and the Supplementary Files S1–S6.

## Supplementary Materials

The supporting information can be downloaded at: https://www.mdpi.com/article/10.3390/ijms232012417/s1.
