# Supplementary figures and images for: Transcriptomic Analysis in Marine Medaka Gill Reveals That the Hypo-Osmotic Stress Could Alter the Immune Response via the IL17 Signaling Pathway
Source: Int J Mol Sci. 2022 Oct 17;23(20):12417. doi: 10.3390/ijms232012417 (PMC9604416; doi:10.3390/ijms232012417)

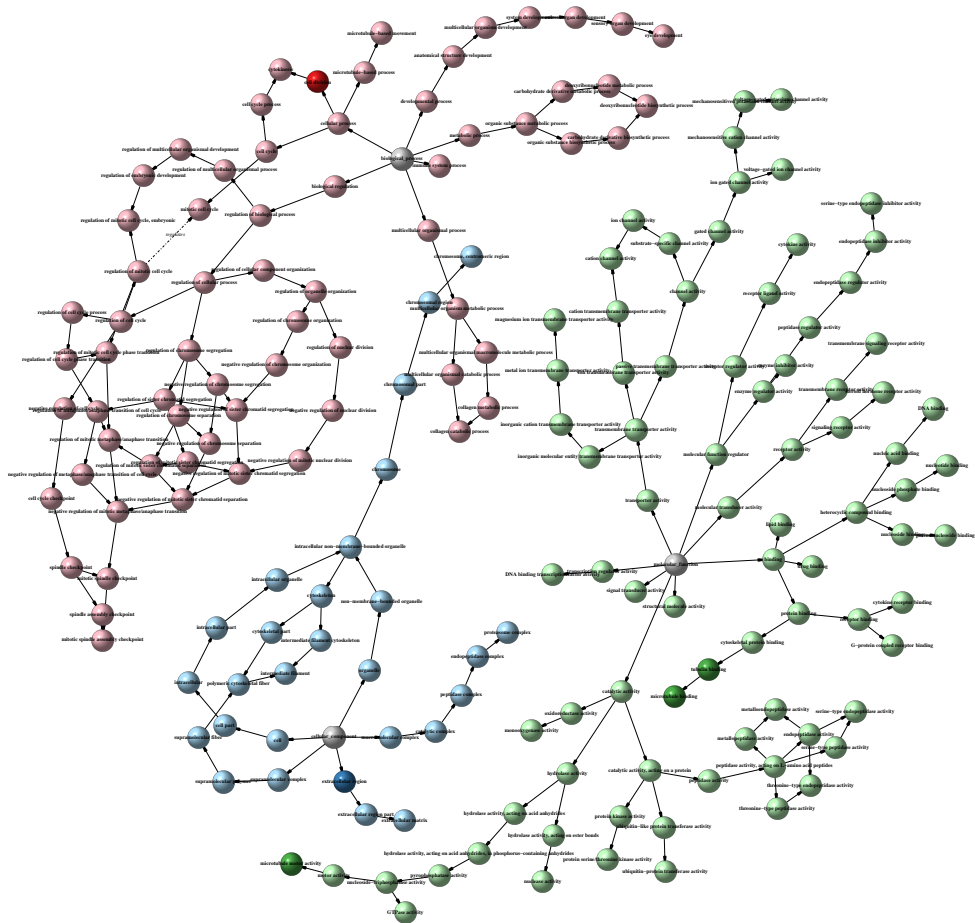

Supplement: Supplementary file 1 [file ijms-23-12417-s001.zip › ijms-1885953-supplementary/Supplementary File S4.pdf]
